# Effectiveness of Magnetic Resonance Angiography in Pre-Operative Assessment of Four Uterine Artery Variants in Women with Symptomatic Uterine Fibroids

**DOI:** 10.14789/ejmj.JMJ25-0048-OA

**Published:** 2026-02-06

**Authors:** HITOMI KATO, JULIANA YUMI ISHISAKI, YOSHIKI KUWATSURU, MASAFUMI ARAI, NAOKI TAKEMASA, RYOHEI KUWATSURU

**Affiliations:** 1 Department of Radiology, School of Medicine, Juntendo University, Tokyo, Japan; 1 Department of Radiology, School of Medicine, Juntendo University, Tokyo, Japan; 2 Department of Radiology, Graduate School of Medicine, Juntendo University, Tokyo, Japan; 2 Department of Radiology, Graduate School of Medicine, Juntendo University, Tokyo, Japan

**Keywords:** uterine artery embolization, uterine artery, magnetic resonance angiography, aortography, leiomyoma

## Abstract

**Objective:**

To evaluate the effectiveness of non-contrast-enhanced magnetic resonance angiography (NCE-MRA) for assessing uterine artery (UA) anatomy and report the prevalence of anatomical forms of the UA in women undergoing uterine artery embolization (UAE).

**Design:**

Retrospective study.

**Methods:**

NCE-MRA images of 110 patients undergoing UAE for the treatment of symptomatic uterine fibroids were retrospectively assessed and the bifurcation patterns of their UAs were bilaterally classified into four types.

**Results:**

In our population, 61% of the UAs presented with type I bifurcation, followed by type IV (16%), type III (15%), and type II (8%).

**Conclusion:**

Preprocedural NCE-MRA allowed for accurate classification of the bifurcation patterns of UAs.

## Introduction

Uterine artery embolization (UAE) is frequently performed to treat symptomatic uterine fibroids. The uterine artery (UA) exhibits various bifurcation patterns. Preprocedural understanding of the anatomy of the UA can facilitate safe and successful catheterization^1-5)^. Gomez-Jorge et al.^1)^ evaluated more than 500 UAs by using internal iliac arteriography, an invasive procedure. Four bifurcation patterns involving the origin of the UA were identified^1)^.

Three-dimensional (3D) non-contrast-enhanced magnetic resonance angiography (NCE-MRA) is a non-invasive modality that allows interventional radiologists to delineate the origin of the UA and visualize its 3D configuration^6)^. This study aimed to examine the effectiveness of NCE-MRA for classifying UA anatomy before UAE and report the anatomical forms of UAs in women undergoing angiography and UAE in our department. The accuracy of NCE-MRA was confirmed using angiography, the standard imaging technique. The anticipated results may promote the utilization of preprocedural NCE-MRA, thereby shortening the catheterization time and minimizing the occurrence of catheter-related injuries.

## Material and Methods

This retrospective study was conducted in accordance with the principles of the Declaration of Helsinki and approved by the Institutional Review Board of Juntendo University Hospital (No. 20-392); informed consent was waived. From December 2016 through November 2020, 110 consecutive patients (mean age 45 y; range 36-52 y) underwent preprocedural NCE-MRA, angiography, and UAE in our department for the treatment of symptomatic uterine fibroids and were included in this research.

Preprocedural NCE-MRA images of each patient retrieved from the hospital’s picture archiving and communication system were reviewed in consensus by two radiologists with 36 and 10 years of experience in female pelvic imaging, respectively. UAEs were performed by an interventional radiologist with 10 years of experience.

### MR protocol and NCE-MRA

From October 2016 through November 2018, a 1.5-T MR unit (Excelart Vantage powered by Atlas; Canon Medical Systems, Japan) was used for image acquisition in our department. The MR machine was replaced with a 3-T unit (Vantage Galan 3T / ZGO; Canon Medical Systems) in December 2018 as previously described^7)^.

Briefly, the pair of phased-array coils (16 channels with 16 elements: Atlas Speeder Body coil combined with Atlas Speeder Spine coil; Canon Medical Systems) were placed at the front and back of the abdomen during imaging.

The combination of true steady-state free precession technique and time-spatial labeling inversion pulse was utilized to obtain 3D NCE-MRA reconstructions of the pelvic vasculature and visualize the UAs, as previously described^7)^.

### Visual classification of the uterine arteries

**Figure 1:**
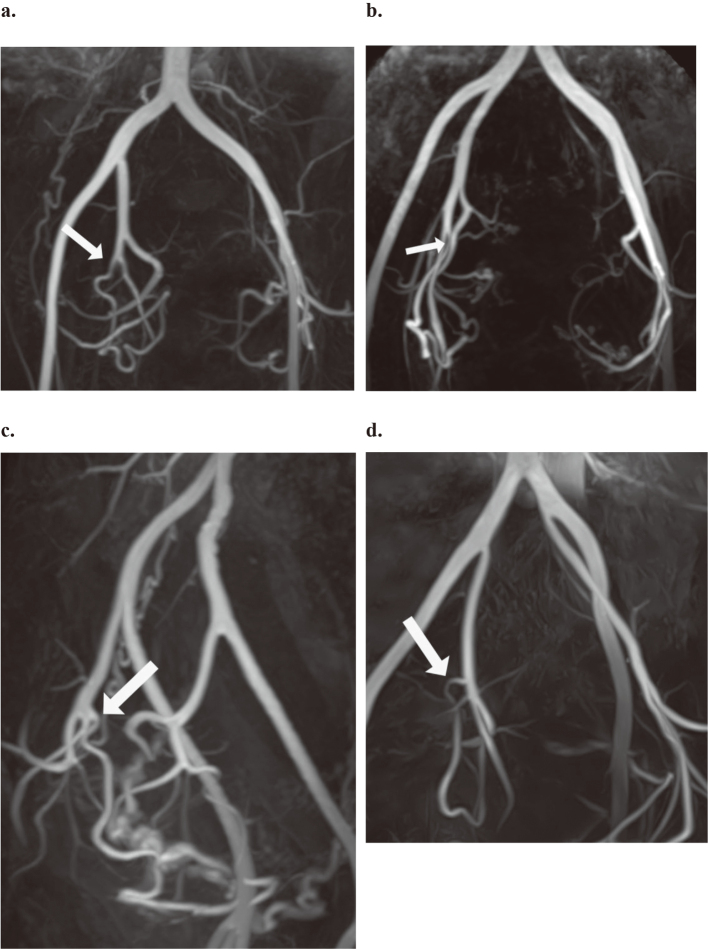
Non-contrast-enhanced magnetic resonance angiography images. (a) The image of a 47-year-old woman with a type I variant. The right uterine artery (arrow) originates as the first branch of the inferior gluteal artery. (b) The image of a 47-year-old woman with a type II variant. The right uterine artery (arrow) arises as the second branch of the inferior gluteal artery. (c) The image of a 49-year-old woman with a type III variant. The right uterine artery (arrow) bifurcates at the same level as the superior and inferior gluteal arteries. (d) The image of a 48-year-old woman with type IV variant. The right uterine artery (arrow) arises as the first branch of the internal iliac artery.

Bifurcation patterns of bilateral UAs were classified into the four types based on the origin, proposed by Gomez-Jorge et al.^1)^. The types of uterine artery origination were: type I: origination from the first branch of inferior gluteal artery after branching from the superior gluteal artery (Figure 1a); type II: from the second or third branch of inferior gluteal artery after branching from the superior gluteal artery (Figure 1b); type III: from trifurcation of UA, internal pudendal artery and inferior gluteal artery of the anterior division (Figure 1c); and type IV: from the first direct branch of the internal iliac artery (Figure 1d).

After the bifurcation patterns were identified by NCE-MRA, abdominal aortic images were taken when UAE was performed, to verify the classification. Contralateral oblique views of the internal iliac arteries were obtained when bifurcation patterns were poorly visualized on abdominal aortic images. Internal iliac artery imaging was generally omitted following preprocedural NCE-MRA, except in technically challenging cases.

## Results

Good quality preprocedural NCE-MRA images from 110 patients were reviewed, and 219 UAs were carefully assessed. In one patient, the left UA was absent. All UAs were cannulated. Among 109 patients with bilateral UAs, 57 presented with different classifications on the right and left sides, and the remaining 52 presented with the same type on both sides (41 with type I; 4 type II; 2 type III, and; 5 type IV). Type I was the most prevalent in our study, accounting for 61% (134 UAs) of the cases. Type II was the least frequently detected pattern (8%), found in 17 UAs. Type III was present in 15% (33 UAs), and type IV was seen in 16% (35 UAs). Abdominal aortic images and oblique internal iliac angiographic images revealed the results were comparable to the NCE-MRA classification.

In this series, there were no other anatomic variants such as replaced small arterial branches, congenital absence of bilateral uterine arteries, or aberrant uterine arteries originating from the abdominal aorta.

## Discussion

We previously demonstrated the effectiveness of NCE-MRA for visualization of UAs^7)^. The present study further demonstrated that NCE-MRA of the UAs allowed accurate assessment of the branch type in all cases analyzed without reference to abdominal aortography or oblique internal iliac angiography images. Anatomic variations of the UA have been previously explored with controversial findings^5)^. Notably, the present results are superior to the results of the previous study by Gomez-Jorge et al.^1)^, which used only internal iliac arteriography images and found that only 38% of the UAs were classifiable. In addition, the high prevalence of type I UAs in the present study is consistent with that in the Gomez-Jorge study, although our incidence of 61% was higher than the 45% reported previously.

The prevalence of type II was similar: 8% in our study and 6% in the Gomez-Jorge study.

The prevalence of type III differed markedly. In our study, 15% of the UAs were classified as type III, compared to 43% in the Gomez-Jorge study. Lastly, type IV UAs accounted for 16% in the present study and 6% in the Gomez-Jorge study, respectively (Table 1).

**Table 1:** Comparison between our findings and those of Gomez-Jorge et al.

Uterine artery classification	The present study	Gomez-Jorge et al^1)^
Type I	134 (61%)	88 (45%)
Type II	17 (8%)	12 (6%)
Type III	33 (15%)	84 (43%)
Type IV	35 (16%)	12 (6%)

The discrepancies in incidence of each type between the present study and the study of Gomez- Jorge might be explained by two factors. Our study was conducted in Japan, whereas the Gomez-Jorge study was conducted in the United States. Both studies likely reflect the different general populations of their respective countries. Second, identification of the uterine artery bifurcation type, by internal iliac arteriography, may be obscured when pelvic arteriography is used. The NCE-MRA technique^7)^ used in the present study produces high-quality images that show the UA and other arteries originating from the internal iliac artery. Because these images are 3D, rotation of the images allows clear differentiation of the arteries originating from the internal iliac artery. Based on our experience with the UAE technique, understanding the uterine artery anatomy prior to the procedure is highly valuable for selective catheterization of the target vessels. Type IV bifurcation, in which the UA arises directly from the internal iliac artery, is relatively easier to operate on than the trifurcation type (type III). In type IV, the UA is the first branch arising from the internal iliac artery. Once the catheter is placed in the right internal iliac artery, the operator locates the UA, which lies between the orifice of the internal iliac artery and the bifurcation of the superior and inferior gluteal arteries. Rotating the NCE-MRA images before UAE shows the orientation of the UA orifice so that the anterior- posterior and medial-lateral direction of catheter advancement can be anticipated. In contrast, for type III, there are three branches arising together. Consequently, in addition to placing the catheter in the correct internal iliac artery, the insertion must be carefully chosen, because there are two vessels other than the target nearby. Preprocedural NCE- MRA is again useful to insert the catheter precisely and quickly.

Therefore, we believe that performing UAE without prior knowledge of the UA anatomical variant can complicate catheterization, prolong procedure time, increase radiation exposure, and increase the risk of UA spasm. We intend to evaluate this hypothesis in a future study. A second advantage of knowing the UA bifurcation type beforehand is that we can take fewer images during the procedure, thereby reducing the radiation exposure; in our hospital, we do not take images of the internal iliac arteries when preprocedural NCE-MRA has been used except in difficult cases. In the present study, the one case of unilaterally absent uterine artery identified on preprocedural NCE-MRA was confirmed by internal iliac arteriography. Although no other anatomic variants were observed in our series, future studies should investigate how such variants appear on NCE-MRA.

This study has several limitations. It was a retrospective analysis conducted at a single institution. Therefore, larger, multi-institutional studies are needed to confirm differences in the prevalence of UA anatomical forms across diverse populations, and to clarify the underlying causes of these variations. Additionally, NCE-MRA was not compared with conventional internal iliac arteriographic images in all cases. In our practice, to minimize radiation exposure, oblique imaging is restricted to cases of difficult catheterization.

In conclusion, our study demonstrated that NCE- MRA yielded clear images of UA anatomy that were classifiable in every case. In our series, type I was the most prevalent variant, while type II was the least common. The prevalence of types III and IV in our cohort in Japan differed markedly from those reported in the U.S. Further studies are needed to confirm these findings and explore potential explanations.

## Author contributions

Conceptualization, RK, HK; methodology, RK, YK, and MA; validation, JYI, MA, and HK; formal analysis, RK, and YK; investigation, YK, MA, and NT; data curation, YK; writing—original draft preparation, HK and YK; writing—review and editing, RK; visualization, RK and YK; supervision, RK. All authors have read and agreed to the published version of the manuscript.

## Conflicts of interest statement

Ryohei Kuwatsuru is on the advisory board of Juntendo Medical Journal and was not involved in the peer review or decision-making process. The other authors declare that there are no conflicts of interest.

## Ethical statement

This retrospective study was conducted in accordance with the principles of the Declaration of Helsinki and approved by the Institutional Review Board of Juntendo University Hospital (No. 20-392)

## Informed consent statement

Informed consent was waived by the Institutional Review Board of Juntendo University Hospital.

## Data availability statement

Data supporting the findings of this study are available from the corresponding author upon reasonable request.

## References

[1] Gomez-Jorge J, Keyoung A, Levy EB, Spies JB: Uterine artery anatomy relevant to uterine leiomyomata embolization. Cardiovasc Intervent Radiol, 2003; 26: 522-527. 15061175 10.1007/s00270-003-2652-7

[2] Pelage JP, Cazejust J, Pluot E, et al: Uterine fibroid vascularization and clinical relevance to uterine fibroid embolization. Radiographics, 2005; 25 Suppl 1: S99-117. 16227501 10.1148/rg.25si055510

[3] Albulescu D, Constantin C, Constantin C: Uterine artery emerging variants - angiographic aspects. Curr Health Sci J, 2014; 40: 214-216. 25729609 10.12865/CHSJ.40.03.11 PMC4340444

[4] Kato H, Kuwatsuru R: Anatomy of Uterine Blood Supply and the Prevention of Massive Hemorrhage. In: Takeda S, Kuwatsuru R, eds. Gynecologic and Obstetric Prophylactic Hemostasis by Intra-arterial Balloon Occlusion. Singapore: Springer Singapore, 2018: 1-6.

[5] Liapis K, Tasis N, Tsouknidas I, et al: Anatomic variations of the Uterine Artery. Review of the literature and their clinical significance. Turk J Obstet Gynecol, 2020; 17: 58-62. 32341832 10.4274/tjod.galenos.2020.33427 PMC7171538

[6] Maciel C, Tang YZ, Sahdev A, Madureira AM, Vilares Morgado P: Preprocedural MRI and MRA in planning fibroid embolization. Diagn Interv Radiol, 2017; 23: 163-171. 28163256 10.5152/dir.2016.16623 PMC5338584

[7] Ishisaki JY, Kato H, Zhang X, et al: Comparison of 1.5 T and 3 T non-contrast-enhanced MR angiography for visualization of uterine and ovarian arteries before uterine artery embolization. Eur Radiol, 2022; 32: 470-476. 34195889 10.1007/s00330-021-08141-z

